# Instance-based error correction for short reads of disease-associated genes

**DOI:** 10.1186/s12859-021-04058-y

**Published:** 2021-06-02

**Authors:** Xuan Zhang, Yuansheng Liu, Zuguo Yu, Michael Blumenstein, Gyorgy Hutvagner, Jinyan Li

**Affiliations:** 1 Advanced Analytics Institute, Faculty of Engineering and IT, University of Technology Sydney, Ultimo, NSW 2007 Australia; 2 Key Laboratory of Intelligent Computing and Information Processing of Ministry of Education and Hunan Key Laboratory for Computation and Simulation in Science and Engineering, Xiangtan University, Xiangtan, 411105 China; 3 Faculty of Engineering and IT, University of Technology Sydney, Ultimo, NSW 2007 Australia

**Keywords:** Error correction, Instance-based method, Illumina reads

## Abstract

**Background:**

Genomic reads from sequencing platforms contain random errors. Global correction algorithms have been developed, aiming to rectify all possible errors in the reads using generic genome-wide patterns. However, the non-uniform sequencing depths hinder the global approach to conduct effective error removal. As some genes may get under-corrected or over-corrected by the global approach, we conduct instance-based error correction for short reads of disease-associated genes or pathways. The paramount requirement is to ensure the relevant reads, instead of the whole genome, are error-free to provide significant benefits for single-nucleotide polymorphism (SNP) or variant calling studies on the specific genes.

**Results:**

To rectify possible errors in the short reads of disease-associated genes, our novel idea is to exploit local sequence features and statistics directly related to these genes. Extensive experiments are conducted in comparison with state-of-the-art methods on both simulated and real datasets of lung cancer associated genes (including single-end and paired-end reads). The results demonstrated the superiority of our method with the best performance on precision, recall and gain rate, as well as on sequence assembly results (e.g., N50, the length of contig and contig quality).

**Conclusion:**

Instance-based strategy makes it possible to explore fine-grained patterns focusing on specific genes, providing high precision error correction and convincing gene sequence assembly. SNP case studies show that errors occurring at some traditional SNP areas can be accurately corrected, providing high precision and sensitivity for investigations on disease-causing point mutations.

## Background

The rapid development of high-throughput next-generation sequencing (NGS) platforms has produced massive sets of genomic reads under low costs for a wide range of biomedical applications [1–4]. Serious concern over these datasets is that there are lots of random errors (such as substitutions, insertions and deletions) existing in these reads. The most popular Illumina platforms generate sequencing data with 0.5–2.5% error rates [5]. Substitutions are the major error type in the short sequencing reads, while insertions and deletions are the major error types in the long sequencing reads.

To avoid possible negative effects on the downstream analysis caused by the sequencing errors, correction algorithms have been previously studied and many tools [6–14] have become available to rectify errors in the raw data. These methods take a global approach to rectify all possible errors using genome-wide patterns and statistics. Because the correction is operated on the whole set of reads (usually millions or billions in number), the algorithm complexity is high and the correction performance is not perfect; sometimes even a lot of new errors are introduced into the reads by these global approaches. These challenges are attributed to several reasons. Firstly, the sequencing depth is non-uniform—the sequencing coverage varies remarkably from one part to another in the genome. The resulting conflicts between the *k*-mer statistics from the low-coverage regions and those from the high-coverage regions have significantly hindered the global approach to conduct effective error removal—Some genes may get under-corrected while some other genes get over-corrected. Secondly, genome fragmentation for read generation is random and the errors are distributed non-uniformly. Thirdly, repetitive regions exist in the genome sequences. Reads from the repetitive regions are likely to share the same nucleotide sequence, or highly similar to each other  [15]. Errors in these reads tend to be corrected falsely by the global approaches and many new errors are introduced.

It is sometimes unnecessary to conduct global correction. Instead, highly-accurate instance-based error correction for short reads of specific genes is more important. For example when SNP [16] or genotyping properties [17] are of great importance, then only specific genes or pathways involved in the disease mechanism or a special segment of loci in the genome would be focused on. In these important situations, the paramount requirement is to ensure the relevant reads, instead of the whole genome, are error free after the correction step. As in a recent breast cancer study [18], the tumour suppressor gene BRCA1 and particularly the single-nucleotide variants (SNVs) in this gene’s exons are focused on understanding the functionally critical domains of BRCA1 and the related clinically actionable genes [19]. It is vital to provide error-free reads related to these specific genes [20] for the precise detection of SNVs and accurate discovery of SNPs. As another example in the mutation and protein research area, error correction is important because one or two DNA base mutations in the coding region of a gene may lead to functionally different amino acids [21–23], and more likely when the open reading frame mechanism is considered. These mutations are called *point* mutations, and more than 31,000 such mutations in the human genome are associated with genetic diseases [24]. The reads related to such a gene without error correction or with under-correction may mislead the conclusion about the functional properties of the proteins. The existing global error correction is not the best choice for this.

In this work, we propose to use an instance-based approach to make error correction for the reads of a disease-associated gene. The method is also applicable to the reads of multiple disease genes, or a set of genes related to a phenotype, or an unknown-function region in the genome, or even any nucleotide sequence of interests. The method, named InsEC, aims to rectify the errors in the instance reads with a very high accuracy and to reduce the number of introduced new errors to a minimum. The global approaches suffer from the issue of non-uniform sequencing depths occurred in error correction. However, when the instance-based approach is taken for the error correction in a subset of reads, this issue can be significantly moderated. Comparing with the global approaches which may have neglected the local features of the instance reads, our instance-based approach has the advantage that the patterns and statistics can be exhaustively explored to rectify the errors, and can be conservatively combined to reduce the number of introduced errors. InsEC has two steps. The first step is for read extraction, which collects all reads relevant to a given gene. The second step is for correction, which exploits the local sequence features in the extracted read sets It uses local alignments to quantify erroneous probability of each base in the reads for an accurate correction.

In fact, global approaches can be turned into instance-based approaches if the whole set of reads is narrowed down to the subset of reads of a specific gene as input data. These global approaches include *k*-mer based error correction methods such as BFC [9], BLESS [11], Lighter [8], Blue [12], and ACE [7]. The key idea of these methods is to use the frequencies of all *k*-mer strings and a global frequency threshold to define solid and weak *k*-mers. The error correction process is to transform each weak *k*-mer into a solid *k*-mer according to some heuristics (e.g., the minimum edit distance between a weak and a sold k-mer). Because the sequencing depths are non-uniform across the genome, some globally weak *k*-mers are actually solid *k*-mers in a local region. Thus it is a wrong correction to transform these local solid *k*-mers. Compared with the global *k*-mer based methods, the global multiple alignment methods, including Coral [13], ECHO [14] and Karect [10], do not rely too much on the selection of *k*-mers. Firstly, reads are grouped based on whether they share some *k*-mer. Then reads in each group are concatenated to form a long consensus contig, which is assumed error-free. Then, these consensuses are used as references to correct the mismatches in every read. But, the *k*-mer grouping can intensify the issue of non-uniform sequencing depths in the contigs, i.e., the error-free assumption on the contigs is too strong and biased.

Our instance-based approach InsEC does not need to define solid or weak *k*-mers in the correction step, and thus it can avoid the issue of non-uniform sequencing depths in the global approaches. Although similarly as the multiple sequence alignment methods to implement the alignment process, our InsEC quantifies error probabilities conservatively column-by-column and row-by-row in the alignment array to avoid introducing new errors.

The performance of InsEC is evaluated on the error correction itself as well as on the quality of the resulted assemblies. Extensive experiments demonstrated that our method has superior precision, recall and gain rates over all state-of-the-art error correction methods when tested on reads datasets of lung cancer associated genes. The quality of the assemblies of the reads also become improved after our error correction. We obtained longer and less number of contigs, and the contigs are closer to the ground truth in the simulated datasets. In our SNP case studies, we found that some corrections can happen at the current lung cancer SNP database, implying that instance-based error correction is crucially important for SNP and mutation analysis.

## Results

We compare the error correction performance of InsEC with instantialized state-of-the-art tools Bcool [6], BFC [9] and Coral [13]. Bcool is the latest method published in year 2020; BFC and Coral are two classical error correction methods, representing the *k*-mer based methods and the multi-alignment error correction methods respectively. Our experiments are conducted on both simulated and real sequencing data. The ground truth of the genome sequence is not available for the real datasets, so the simulated datasets are used as a supplement to the real data experiments. With the ground truth provided by the simulated datasets, we are able to evaluate error correction and further assembly performance objectively for all of the methods. Our InsEC method is designed for error correction on disease-causing genes, so seven genes related to lung cancers are selected to illustrate method performance in the following experiments.

### Extracted read datasets of lung cancer associated genes

Illumina sequencing datasets are available at the Sequence Read Archive (SRA) (https://www.ncbi.nlm.nih.gov/sra/); and the simulated Illumina sequencing data can be produced by ART [25] which is a benchmark tool for the generation of simulated short reads. The real dataset used in this work is ERR174310, which contains paired-end human whole genome deep sequencing reads generated by Illumina HiSeq 2000. We denote this dataset as D0. The two simulated sequencing datasets (denoted by D1 and D2) have the same read length and the same sequencing platform as ERR174310. D1 is a single-end dataset, and D2 is a paired-end dataset, both generated with reference to the standard sequence of human chromosome one. The genome annotations are obtained from the NCBI (National Center for Biotechnology Information) (https://www.ncbi.nlm.nih.gov/genome/), including gene name, gene ID and gene positions. More details of these datasets are shown in Table 1.

**Table 1:** Description of the datasets

Dataset	Real dataset	Simulated dataset	
	D0	D1	D2
Read length	100	100	100
Total reads	586,941,413	23,046,123	23,048,001
Type of reads	Paired-end	Single-end	Paired-end
Accession No.	ERR174310	Simu-Single	Simu-Pair
Reference	Human Genome	Chromosome.1	Chromosome.1

The latest version of human genome, GRCh38.P13, is used in our experiments as of September 2019

The seven genes related to lung cancer in this study are ILR6R, IL10, ATF3, GRIK3, MYCL, PRDX1, and ENO1. All of these genes are located at chromosome one. The nucleotide sequences of the genes are available at the NCBI gene database (https://www.ncbi.nlm.nih.gov/gene/). The length of these genes ranges from 4,892 to 238,602 bases. See more details of these genes in Table 2.

**Table 2:** Genes related to lung cancer on human chromosome one

Gene_ID	Gene_Name	Gene_length	Gene_function
Gene1 (g1)	IL6R	64257	protein_coding
Gene2 (g2)	IL10	4892	protein_coding
Gene3 (g3)	ATF3	55443	protein_coding
Gene4 (g4)	GRIK3	238602	protein_coding
Gene5 (g5)	MYCL	6830	protein_coding
Gene6 (g6)	PRDX1	12011	protein_coding
Gene7 (g7)	ENO1	18250	protein_coding

The details of genes are from the genome annotation of the latest version GRCh38.P13

### Performance evaluation metrics

The performance is evaluated not only on the error correction but also on the read assembly before and after the error correction.

#### Metrics for error correction performance

To assess the accuracy of the correction methods, we use the following three metrics.

- Precision: *TP/(TP+FP)*, shows the fraction of truly corrected bases among all changed bases.
- Recall: *TP/(TP+FN)*, shows the fraction of truly corrected bases among all bases which are supposed to be corrected.
- Gain: *(TP-FP)/(TP+FN)*, shows the fraction of removing errors without inducing additional errors.

where true positives (TP) correspond to corrected errors; true negatives (TN) correspond to initially correct bases left untouched; false positives (FP) correspond to newly introduced errors; and false negatives (FN) correspond to unidentified errors.

#### Metrics for assembly performance

To assess the impact of error correction on the assembly results, we compare InsEC with other state-of-the-art methods by standard assembly assessment metrics. We choose SPAdes [26] to assembly read data before and after error correction, except that the error-free datasets are assembled for the performance assessment as well. To assess our method more specificlly, each nucleotide in the gene sequence updated by InsEC is compared with its in gene reference. On simulated dataset, the ground truth of gene sequence is available, so the more similar the updated sequence with the referferce is, the better performance of assembly is.

- Assembly results comparison: the assembly results are evaluated by QUAST [27], a quality assessent tools for genome assemblies. Detailed reports include the number of contigs, the largest contigs and N50. A contig is a continuous nucleotide sequences obtained from the assembly process. N50 is defined as the minimum contig length needed to cover 50% of genome.
- The Reference vs the corrected sequence: The nucleotide of gene sequences, updated by our method, are compared with the reference sequence of genes base-by-base. The less difference between the two sequences is, the better assembly performance is.

### Performance by instance-based error correction and comparison with state-of-the-art methods

For each *g* of the seven lung cancer disease-associated genes, we constructed $subset(D1, I_g)$ and $subset(D2, I_g)$, and conducted instance-based error correction by InsEC. Strictly on these two subsets of reads, we also apply three state-of-the-art global correction methods Bcool [6], BFC [9] and Coral [13] to rectify errors for a fair comparison. This is exactly so called “global approaches can be turned into instance-based approaches” as stated in Introduction. The overall error correction performance by InsEC, Coral, BFC and Bcool on the seven lung cancer disease genes are presented in Table 3.

**Table 3:** Performance comparison of instance-based error corrections

	On single-end reads				On paired-end reads			
	Ins_EC	Coral	BFC	Bcool	Ins_EC	Coral	BFC	Bcool
Precision ($\%$)								
g1	**98.42**	95.95	91.91	93.01	**99.49**	94.46	90.96	89.82
g2	**100**	99.65	**100**	94.70	**100**	97.39	**100**	98.18
g3	**99.64**	92.19	93.90	95.48	**99.85**	93.49	94.10	97.97
g4	**99.93**	94.86	97.34	96.30	**99.97**	95.19	98.18	98.00
g5	**100**	**100**	**100**	98.68	**100**	90.16	95.00	96.02
g6	**98.56**	93.36	91.64	87.73	**99.27**	92.30	95.35	91.49
g7	**100**	99.25	99.87	93.79	**100**	98.57	96.02	95.81
**AVE**	**99.51**	96.47	96.38	94.24	**99.80**	94.51	95.66	95.33
Recall ($\%$)								
g1	**95.06**	91.06	78.64	79.38	**96.78**	93.92	95.04	86.78
g2	**97.26**	95.65	71.91	89.63	**99.32**	97.39	95.93	96.42
g3	**98.07**	97.07	76.00	89.23	**98.48**	97.92	95.49	92.75
g4	**97.16**	96.97	78.19	91.25	**97.82**	97.05	97.75	93.85
g5	**99.34**	61.84	69.74	98.03	**99.78**	90.16	94.44	95.73
g6	**99.60**	96.44	76.91	79.34	**99.69**	97.20	96.65	81.10
g7	**99.72**	96.81	71.52	86.53	**99.87**	98.41	95.48	89.78
**AVE**	**98.03**	90.83	76.13	87.63	**98.82**	96.01	95.83	90.91
Gain ($\%$)								
g1	**93.54**	87.95	71.72	79.38	**96.29**	89.66	85.60	86.78
g2	**97.26**	95.64	71.91	89.63	**99.32**	95.71	95.93	96.42
g3	**97.71**	89.56	71.06	89.23	**98.34**	92.15	89.50	92.75
g4	**97.09**	92.76	76.06	91.25	**97.79**	93.29	95.94	93.85
g5	**99.34**	61.84	69.74	98.03	**99.78**	81.04	89.47	95.73
g6	**98.15**	91.32	69.90	79.34	**98.96**	91.23	91.94	81.10
g7	**99.72**	96.79	71.43	86.53	**99.87**	98.39	91.52	89.78
**AVE**	**97.55**	87.98	73.11	87.63	**98.62**	91.64	91.42	90.91

AVE indicates the average score over the seven genes. Bold font indicates the best result in the row

Our method InsEC achieved the best precision, recall and gain rate on all of the datasets. In particular, the average precision, recall and gain rate by our method are much superior respectively by 3.13%, 21.9% and 24.44% to the latest method Bcool on the single-end datasets, and much superior respectively by 4.14%, 2.99% and 7.2% on the paired-end datasets. More importantly, our method improved the gain rates a lot, implying more number of bases are rectified and less number of errors are induced compared with the existing methods. In detail, InsEC improved the gain rates ranging from 9.57% to 24.44% on the single-end datasets, and improved the gain rates ranging from 6.98% to 7.71% on the paired-end datasets. It is noted that the other methods are sensitive to data types. All of the other methods perform better on pair-end datasets than single-end datasets, especially the gain rate improved from 3.28% to 18.31%. While our method InsEC shows good robustness on both single-end and pair-end datasets, achieving the gain rate at 97.55% and 98.62% respectively.

All the experiments were conducted on a computing cluster running Red Hat Enterprise Linux 6.7 (64 bit) with Intel Xeon E5-2695 v3 and 128 GB RAM. We use the Linux/Unix time command to record the system time and memory usage. The average running time (seconds) of InsEC, Coral, BFC and Bcool is 3.2 s, 1.55 s, 1.02 s and 18.92 s and the average memory usage (kbytes) is 503,271 kb, 419,156 b, 1,109,266 kb and 527,268 kb respectively. Our InsEC ranks the second in running time and memory usage.

### The global approaches improved when focusing on disease-associated genes

To show the significance of instance-based error correction for the reads related to disease-causing genes, we compare the error correction performance on the whole sequencing datasets with those on the gene-related subsets of reads.

After running error correction on the whole datasets D1 and D2, those reads relevant to the given gene *g* are extracted for performance assessment and comparison. The methods are specially denoted as Bcool_g, BFC_g and Coral_g in this situation. The overall error correction performance for lung cancer-associated genes is presented in Table 4.

**Table 4:** Performance comparison. Instance-based approach vs global approach

	On single-end reads						On paired-end reads					
	Coral_g	Coral	BFC_g	BFC	Bcool_g	Bcool	Coral_g	Coral	BFC_g	BFC	Bcool_g	Bcool
Precision ($\%$)												
g1	97.80	95.95	85.93	91.91	90.21	93.01	97.97	94.46	92.86	90.96	91.12	89.82
g2	99.66	99.65	94.30	100	94.70	94.70	92.43	97.39	99.00	100	98.18	98.18
g3	98.81	92.19	94.58	93.90	95.48	95.48	98.41	93.49	98.04	94.10	97.32	97.97
g4	98.97	94.86	96.45	97.34	96.30	96.30	98.61	95.19	98.02	98.18	98.00	98.00
g5	98.68	100	100	100	98.01	98.68	96.87	90.16	91.03	95.00	95.02	96.02
g6	93.33	93.36	77.63	91.64	87.73	87.73	92.97	92.30	89.41	95.35	91.49	91.49
g7	99.02	99.25	90.27	99.87	93.79	93.79	95.77	98.57	92.39	96.02	95.81	95.81
**AVE**	98.04	96.47	91.31	**96.38**	93.75	**94.24**	96.15	94.51	94.39	**95.66**	95.28	**95.33**
Recall ($\%$)												
g1	72.48	91.06	75.91	78.64	76.99	79.38	74.61	93.92	92.94	95.04	79.29	86.78
g2	96.99	95.65	82.94	71.91	89.63	89.63	95.60	97.39	96.26	95.93	96.42	96.42
g3	88.00	97.07	75.62	76.00	89.23	89.23	90.19	97.92	96.65	95.49	92.47	92.75
g4	90.26	96.97	78.03	78.19	91.25	91.25	91.47	97.05	96.76	97.75	93.85	93.85
g5	98.68	61.84	79.61	69.74	97.37	98.03	98.80	90.16	90.67	94.44	94.74	95.73
g6	67.09	96.44	77.04	76.91	79.34	79.34	68.96	97.20	94.00	96.65	81.10	81.10
g7	82.35	96.81	80.16	71.52	86.53	86.53	86.31	98.41	93.78	95.48	89.78	89.78
**AVE**	85.12	**90.83**	78.47	76.13	87.19	**87.63**	86.56	**96.01**	94.44	**95.83**	89.66	**90.91**
Gain ($\%$)												
g1	71.32	87.95	63.48	71.72	68.63	79.38	73.79	89.66	85.79	85.60	71.56	86.78
g2	96.98	95.64	77.93	71.91	84.62	89.63	88.93	95.71	95.28	95.93	94.63	96.42
g3	87.66	89.56	71.29	71.06	85.01	89.23	89.57	92.15	94.72	89.50	89.92	92.75
g4	90.15	92.76	75.16	76.06	87.75	91.25	91.24	93.29	94.81	95.94	91.93	93.85
g5	97.37	61.84	79.61	69.74	95.39	98.03	96.77	81.04	81.73	89.47	89.77	95.73
g6	63.58	91.32	54.85	69.90	68.24	79.34	64.52	91.23	82.87	91.94	73.56	81.10
g7	82.05	96.79	71.52	71.43	80.80	86.53	83.40	98.39	86.05	91.52	85.86	89.78
**AVE**	84.16	**87.98**	70.55	**73.11**	81.49	**87.63**	84.03	**91.64**	88.75	**91.42**	85.32	**90.91**

AVE indicates the average score over the seven genes. Bold font indicates the better result compared methods with its _g version

These global error correction methods got improved when directly applied to the subsets of reads related to the gene-associated genes, namely the gain rates by Coral, BFC, and BCOOL are better than their global versions (labeled with _g), increasing the performance from 2.56 to 7.61%.

### Performance of read assembly after error correction

To see whether the error correction has impact on the quality of the assemblies, we compare on the number of contigs, the longest contigs and N50 before and after the error correction of D1 and D2. We also construct the assemblies from the error-free read sets (the ground truth is available for the simulated datasets). The best error correction method is expected to have the most similar assembly results to those from the error-free dataset. The differences in the assembly results between the error-free datasets and corrected datasets after error correction by all the methods are listed in Table 5. There are no differences in assembly results for the other four genes, so their results are not listed in table.

**Table 5:** Assembly results compared with the ground truth

	g1			g3			g4		
	NO.	Lar.	N50	NO.	Lar.	N50	NO.	Lar.	N50
*Single-end reads*									
Truth	6	24854	11363	3	27822	27822	3	187434	187434
Raw	− 1	3170	− 1316	0	50	13879	− 2	37224	37224
**InsEC**	**0**	**0**	**0**	**0**	**0**	**0**	**0**	− **13**	− **13**
Coral	2	− 11485	0	0	− 50	− 13879	1	15	15
Corel_I	4	− 28181	− 41672	1	− 13824	− 13824	0	− 186	− 186
BFC	− 1	3170	− 1316	0	50	13879	0	34	34
BFC_I	− 2	0	0	0	50	50	0	34	34
Bcool	− 1	3198	− 1316	0	50	13879	− 2	52585	52585
Bcool_I	− 1	0	0	0	− 6	− 6	− 1	52505	52505
*Paired-end reads*									
Truth	3	40458	40458	2	27893	27893	6	134849	134849
Raw	− 4	13097	27287	0	63	63	− 3	15530	15530
**InsEC**	− 1	0	0	**0**	**0**	**0**	**0**	**13**	**13**
Coral	0	0	0	0	91	91	− 2	302	302
Corel_I	2	− 23894	− 23894	1	− 27650	− 27650	2	− 23329	− 23329
BFC	0	0	0	0	91	91	− 2	783	783
BFC_I	− 2	0	0	0	69	69	− 2	813	813
Bcool	**0**	**0**	**0**	0	91	91	− 3	15565	15565
Bcool_I	0	63	63	0	63	63	− 2	64126	70640

Truth row indicates the assembly results of the error-free read data. Other rows show the difference value where value in Truth row minus the current row. NO. indicates the number of contigs. Lar. Indicates the largest length of contigs

Bolf font indicates the best assembly result

The assembly results get improved after the error correction. In particular, there is an increasing trend at the length of contigs after the error correction, and a decreasing trend at the number of contigs. Compared with the other error correction methods, InsEC has the most similar assembly results to those from the error-free datasets for 5 of the 6 cases; on the remaining one, the result of our method has only one difference in the number of contigs. Furthermore, we achieved the identical assembly results as those from the error-free datasets g1, g3 and paired-end g3.

The contig quality are shown in Table 6, where the numbers of base differences between the contigs from our corrected reads and those from the reference sequences are presented. Most of the contigs assembled from the corrected reads by our method are identical to the reference sequences (see the sign ‘M’); while the remaining assemblies have only tiny differences from the reference sequences (e.g., only 7 or 6 base differences over a length of 238,603 bases).

**Table 6:** The contigs from corrected reads vs the reference sequence

Contig_Q	g1	g2	g3	g4	g5	g6	g7
Single-end_D1	6/64258	M	5/55444	7/238603	M	2/12012	M
Paired-end_D2	6/64258	M	5/55444	6/238603	M	M	M

The sign ‘M’ indicates the contig assembled from the corrected reads by our method and the reference sequence are identical. 6/64258 indicates there are 6 different bases in 64258 bases, and similarly for other number combinations

### Case studies: error correction at mutation-prone regions in the lung cancer associated genes

On the real sequencing reads dataset D0, we have performed instance-based error correction for the reads relevant to EGFR and KARS which are two genes highly associated with lung cancer [28]. Some of our corrections happened at the mutation-prone regions of EGFR. These point mutations or mutation combinations are known [29] to make lung carcinomas more responsive to treatments with tyrosine kinase inhibitors. These mutations are usually at least one base different from a reference sequence, also referred to ’variant calling’.

One of the corrections changes A to G at the SNP:rs1476431328 position, located at chr7:55205427. Due to this base correction from A to G, the corresponding amino acid is changed from Asparagine (AAC) to Serine (AGC). If this base is not corrected, the amino acid Asparagine instead of the correct amino acid Serine would be focused in the downstream analysis which may lead to different conclusions about the functions of the protein. This is quite possible because Asparagine and Serine pose their own distinct biophysical properties.

**Fig. 1:**
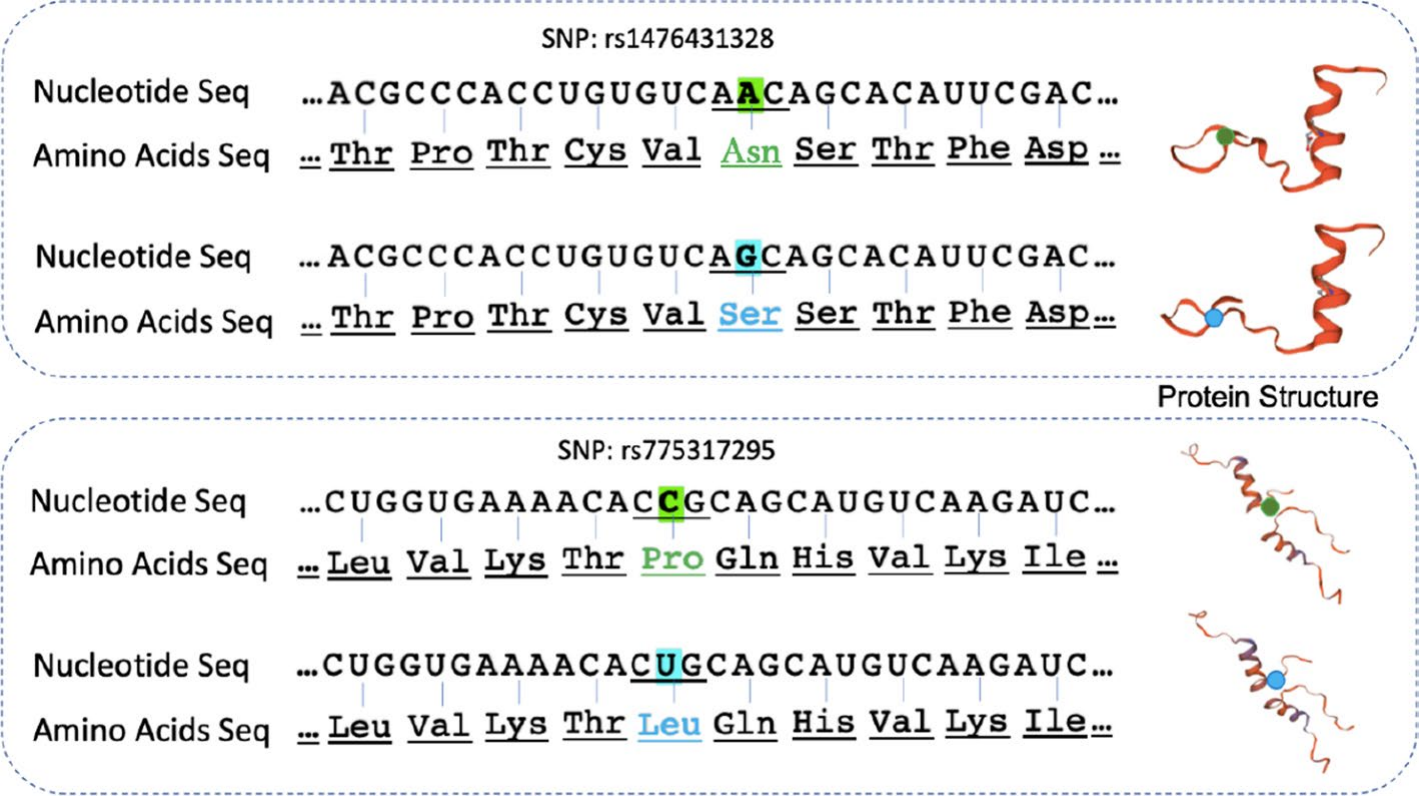
Two examples of point mutations in case studies. The mutation bases and changed amino acids are highlighted by green and blue color. The predicted structure of coding proteins are shown in the right side

Another of our corrections is at SNP:rs781609053 which changes nucleotide T to C. Correspondingly, the amino acid would be changed from Methionine (ATG) to Threonine(ACG). Furthermore a correction was performed at SNP:775317295 which changes nucleotide C to T, implying that the amino acid Proline (CCA) should be changed to Leucine (CTA). The effects of mutations lead to different structures of its coding proteins, thereby affecting its functions  [30], which is shown in Fig. 1, where we use SWISS-MODEL  [31] to model the structure of coding protein according to its amino acids sequence.

The amplification of gene KARS primarily decides the growth and survival of lung cancer cell lines  [32]. For the reads in D0 that are relevant to KARS, some of our instance-based error corrections also occurred at its SNP positions. The correction from A to G at SNP:rs35225896 changes the corresponding amino acid from Isoleucine (ATA) to Methionine (ATG). Highly accurate sequences near this position should be ensured, as mutations at this position are closely related to hereditary cancer-predisposing syndrome, supported by clinical significance and publications (https://www.ncbi.nlm.nih.gov/snp/rs35225896). Error corrections at non-coding regions are important as well. For instance, our correction at SNP:rs11762213 changes the nucleotide from G to A. Though such corrections at non-coding regions do not effect type of amino acids, SNP:rs11762213 is recognized as a predictor of adverse outcomes in clear cell renal cell carcinoma [33]. Thus, high-quality corrections at mutation-prone regions (coding and non-coding regions) are very important for downstream SNP and mutation studies.

## Discussion

Our approach (named InsEC) is contrast to the existing error correction methods which all take a global approach to make a genome-wide error correction. Genome-wide error correction is not good enough especially when the study is focused on disease genes or pathways.

InsEC’s correction step adequately exploits fine-grained local patterns so as to rectify those errors which were unable to be corrected by the global approach. The reason is that the instance-based approach can significantly moderate the global approach’s issue on the non-uniform sequencing depth. We have conducted extensive experiments on simulated single-end and paired-end reads. The performance evaluation confirms that InsEC has much superior precision, recall and gain rate over the state-of-the-art methods on various sets of reads related to lung cancer genes. InsEC can also provide an assembled nucleotide sequence of the corrected reads which is closer to the ground truth than the other methods on the simulated datasets. Our SNP case studies on the real paired-end reads show that the error correction can happen at the mutation-prone bases stored at the current SNP databases, implying that highly accurate instance based approach is particularly useful for SNP and mutation investigations.

## Conclusions

In this work, we have proposed a novel approach for short reads error correction. The method is an instance-based approach, or a local approach, to rectify all possible errors in the reads relevant to a disease gene, or a subset of disease-associated genes. Our novel idea is to exploit local sequence features and statistics directly related to these genes. Two main steps can collects reads relevant to a given gene from a WGS dataset through a noise-tolerant mapping technique and take advantage of alignment processes and rectify errors according to fine-grained patterns and statistics. InsEC achieves good performance on both single-end and pair-end datasets, and can also provide an assembled nucleotide sequence for gene sequence studies. This study successfully serves as read preprocess tools to provide high-quality data for targeted genes or genome region research.

## Methods

A read *r* is a genomic sequence denoted by $r = r_1r_2\cdots r_n$, $r_i \in \Sigma = \{\text{ A }, \text{ C }, \text{ G },\text{ N }, \text{ T }\}$ , where A, C, G and T stand for the nucleotides Adenine, Cytosine, Guanine and Thymine respectively, and the character N stands for uncertain nucleotide; and *n* is the length of *r* (e.g., $n=100$ or 200). Usually, the length of all of the reads from one wet-lab experiment (short read sequencing) is exactly the same. The sequencing errors can be randomly distributed anywhere in *r*.

Computation required by InsEC consists of two main tasks. One task is to draw relevant reads to a given gene from a WGS sequencing dataset. Through read extraction, a gene-related read dataset is constructed for error correction. The second task is to precisely correct errors on the gene-related subset of reads using fine-grained alignment patterns and statistics.

### Reads extraction

Let *S* be a set of human genomic reads generated by Illumina whole genome sequencing platforms, and let $I_g$ be a reference sequence of our interested gene *g*. But the reference sequence $I_g$ is assumed *not* error-free. We extract reads from *S* which are relevant to the gene sequence $I_g$ for the correction of possible errors in these reads. This subset of reads is denoted by $subset(S,I_g)$. We also assume that the ground truth of gene sequence can vary from different individual samples because of single-nucleotide polymorphism. So the ground truth of gene *g*, denoted by $T_g$, should have different nucleotide bases with the reference gene sequence $I_g$. Under the above two assumptions, reads having a Hamming distance with $I_g$ (i.e., with noise tolerance) are required to move from *S* to form $subset(S,I_g)$. The Hamming distance threshold is set as 95 so as to have complete relevance of $subset(S,I_g)$ to $T_g$ as much as possible. In this work, we use BWA-MEM [34] for the read mapping with Hamming distance tolerance. BWA-MEM is a widely-used alignment tool, highly efficient to align short reads against a nucleotide sequence, and it allows mismatches and gaps, which means the extracted subsets of reads may contain insertion and deletion (indel) errors as well. These indel errors are handled at the multiple sequence alignment stage. Insertions are directly removed and the deletions are recovered by the alignment mechanism.

We note that this reads extraction step is very similar to the reads extraction step used in variant calling studies [17, 35]. But the purpose and assumptions are polarly different. The purpose of variant calling studies is to identify variations between genomes and the reference genome is assumed to be error-free. But the purpose of our study is to make corrections for the possible errors in the extracted reads, and the reference genome is assumed to be not error-free. Variant calling studies do not have any attempt to correct the possible errors in the extracted reads. Our error-corrected reads can be used for potentially better variant calling analysis.

In the reads extraction step, we actually extend the sequence $I_g$ at both ends with 50 nucleotide bases, to guarantee that some reads crossing the boundary of $I_g$ can be extracted as well. Through the extension of the gene sequence and the noise-tolerant mapping process, more reads are extracted as far as possible. We note that a few reads mapped to the nucleotide sequence $I_g$ with high mapping scores may belong to other genes (the repetitive areas). So in a further step, we double-check whether a read should be collected in $subset(S,I_g)$.

### Error correction step

After $subset(S,I_g)$ is formed, we align all the reads in $subset(S,I_g)$ according to their positions in $I_g$, and place them one by one in each row in an increasing order of their start position. This sorted organization of $subset(S,I_g)$ is called an alignment array.

The alignment array is traversed column-by-column for error correction. Intuitionally, if a base has a very low type frequency in the column, this base (i.e., an outlier) is very likely to be erroneous. The key idea is to detect dominance information in the columns according to the nucleotide type distribution and to locate error bases in the rows according to their error-aware probabilities.

Suppose only four nucleotide types (i.e., A, C, G, and T) are in the reads. For a column of bases in the alignment array, there are four possible cases for the nucleotide type distribution:

- One-type dominance. All or almost all of the bases have the same nucleotide type. For example, 99% of the bases in the column are nucleotide type ‘A’; all the other bases (‘C’, ‘G’, or ‘T’) constitute the remaining 1% of the bases. These 1% of the bases are outlier bases or erroneous bases.
- Two-type dominance. All or almost all of the bases are split into two main nucleotide types.
- Three-type dominance. All or almost all of the bases are split into three main nucleotide types.
- Four-type dominance. All of the bases are split into four main nucleotide types.

We say a column is dominated by one or more types of bases if the total count of *the other types* of bases is 0, 1, 2, or 3; or the total percentage of the other types of bases is less than 2% when the total number of bases in the column is 100 or more. These thresholds can be adjusted according to data characteristics.

The respective error correction is as follows:

- Correction for one-type dominance. Suppose the dominant type of bases is *X*, then change all other type(s) of base(s) to *X* for correction;
- Correction for two-type dominance. Suppose the two dominant types of bases are *X* and *Y*, then change all other type(s) of base(s) to *X* and *Y* proportional to the percentages of *X* and *Y*;
- Correction for three-type dominance. Suppose the three dominant types of bases are *X*, *Y* and *Z*, then change all other bases to *X*, *Y* and *Z* proportional to the percentages of *X* and *Y* and *Z*;
- Correction for four-type dominance. No correction is needed.

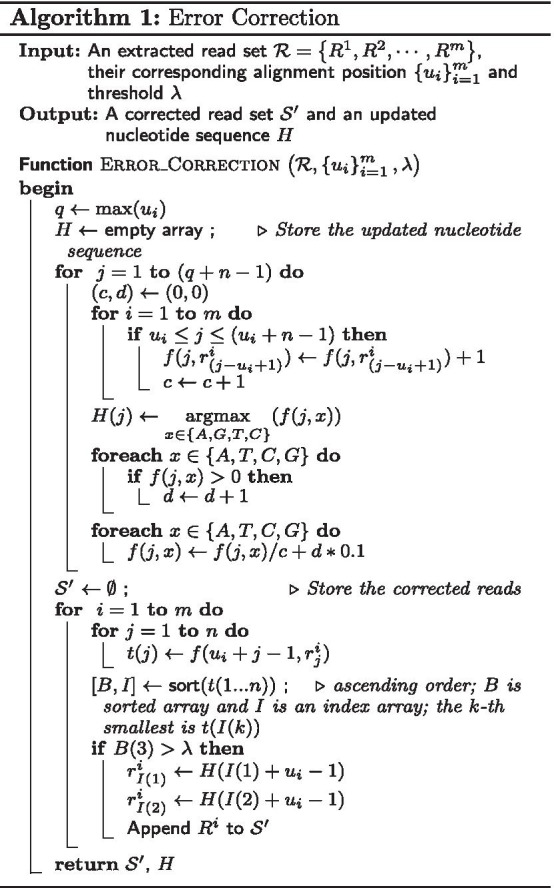


Let *f*(*X*) denote the percentage of *X* in the column, namely the frequency of *X*. Some examples of the base distribution and error correction are: (i) $f(A)=99\%$, $f(T)=0.5\%$, $f(G)=0.5\%$ (dominated by one type), change all the Ts and Gs to A; (ii) $f(T)=40\%$, $f(G)=58\%$, $f(A)=0.8\%$, $f(C)=1.2\%$ (dominated by two types), change all the As and Cs to T and G in the ratio 40:58; $f(T)=40\%$, $f(G)=58\%$, $f(A)=2\%$ (dominated by two types), change all the As to T and G in the ratio 40:58; (iii) $f(T)=40\%$, $f(G)=41\%$, $f(A)=18\%$, $f(C)=1.0\%$ (dominated by three types), change all the Cs to T and G and A in the ratio 40:41:18; $f(T)=40\%$, $f(G)=41\%$, $f(A)=19\%$ (dominated by three types), no change; and (iv) $f(T)=25\%$, $f(G)=40\%$, $f(A)=30\%$, $f(C)=5\%$ (dominated by four types), no change.

If the minor types of the bases have the same frequency at multiple columns, for a conservative correction, we set priorities to change those bases at the columns with a less number of dominant types. The order is: one-type dominance is prior to two-type dominance which is prior to three-type dominance. The priority value of base *V* is set as 0.1 if *V* is at a one-type dominance column, denoted by $p(V)=0.1$; set as 0.2 if *V* is at a two-type dominance column, denoted by $p(V)=0.2$; and set as 0.3 if *V* is at a three-type dominance column, denoted by $p(V)=0.3$.

We then traverse the alignment array row-by-row to make the conservative error correction. For each row, we rank all the bases $r_1r_2\cdots r_n$, according to their base type frequency together with their dominance value (i.e., $f(r_i) + p(r_i)$), into an increasing order. Since Illumina sequencing data (used in this work) has an error rate around $0.5\%$ to $2\%$, the first two per cent of bases in a row are considered as errors. Then these bases are confirmed to change. Before changes, we check the number of dominant types in the column. If there are more than one potential dominant type to correct, we consider its neighbor columns as well. We give a high priority to corrections which is followed by dominant types with large number of bases.

**Fig. 2:**
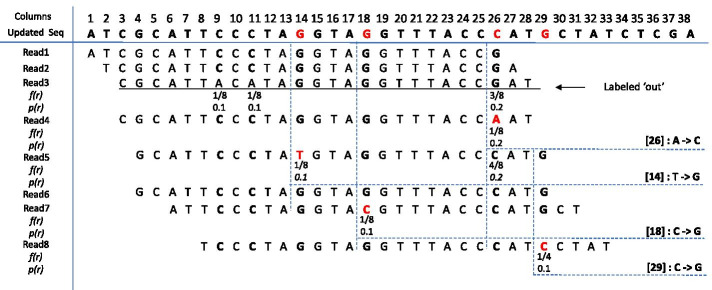
An example of read correction on eight reads. The base-type frequency *f*(*r*) and dominance value *p*(*r*) of a base are shown below that base. For the columns of bases, the dominant nucleotide types are in bold and the erroneous bases are in the red color. The updated sequence is on the top and the correction details are listed in the right. For example, in column 26, there are two dominant nucleotides (*G and C*). *f*(*C*) is larger than *f*(*G*), so the nucleotide C is used to update the sequence, and the erroneous base A (position[26]) in read4 is corrected to C. For read3, the third base after ranking is below the threshold, so read3 is labeled ‘out’ and deleted from the extracted subset

Note that in the situation of two-type or three-type dominance, some of the reads in $subset(S,I_g)$ are not relevant to gene *g*. They may come from another gene with a repetitive region of *g*. This issue is not solvable by the reads extraction step; it is only identifiable in the alignment step. In this work, if more than one of bases’ probability in the top two per cent bases is larger than the threshold, we assume the read are more likely from the other part of the genome sequence *I*, instead of from the sequence of the gene $I_g$. These reads are labeled ’out’ and deleted from $subset(S,I_g)$ for the contig construction of gene *g*. An example of the correction is shown in Fig. 2. The pseudo code of the correction algorithm is shown in Algorithm 1.

## Data Availability

InsEC is available open source under the GNU General Public License v3.0 at https://github.com/XuanrZhang/InsEC. Datasets are available at the same link.

## Abbreviations

SNP: Single-nucleotide polymorphism
NGS: Next-generation sequencing
SNVs: Single-nucleotide variants
SRA: Sequence Read Archive
WGS: Whole genome sequencing
NCBI: National Center for Biotechnology Information
